# Forecasting the use of chiropractic services within the Veterans Health Administration

**DOI:** 10.1371/journal.pone.0316924

**Published:** 2025-01-13

**Authors:** Victoria A. Bensel, Kelsey Corcoran, Anthony J. Lisi

**Affiliations:** 1 Biomedical Informatics and Data Science, Yale School of Medicine, New Haven, CT, United States of America; 2 VA Connecticut Healthcare System, West Haven, CT, United States of America; Endeavour College of Natural Health, AUSTRALIA

## Abstract

**Objective:**

To model future use of chiropractic services and predict clinical resource needs within the Veterans Health Administration (VA) over the next 5 years.

**Methods:**

A serial cross-sectional analysis of chiropractic use data from VA’s Corporate Data Warehouse for fiscal years (FY) 2017 through 2022 (10/1/2016-9/30/2022). We calculated the proportion of VA chiropractic users–via care provided on-station and/or purchased from Community Care Network (CCN) providers–compared to overall VA healthcare users for each FY. We calculated the historical year-over-year compound annual growth rate (CAGR), which was used to predict use in FY2023 through 2027 (10/1/2022-9/30/2027).

**Results:**

VA’s chiropractic use rate increased from 1.4% in FY2017 to 3.5% in FY2022, at which point 2.0% of VA users received only CCN chiropractic care, 1.3% only on-station, and 0.2% both. During the 6-year observation period, the CAGRs were overall 17.9%, CCN only 23.8%, on-station only 12.4%, and both 27.7%. Using those rates to extrapolate, by the end of FY2027 overall use will be 8.9%, with 5.9% only CCN, 2.3% only on-station, and 0.6% both.

**Conclusion:**

Overall use of VA chiropractic services is projected to more than double from FY 2022 to FY2027. These findings underscore the need for proactive resource planning to address the expected increased use of both CCN and on-station care.

## Introduction

Healthcare systems worldwide increasingly rely on predictive models as essential tools to optimize resource allocation, enhance patient outcomes, and bolster the efficiency of healthcare services. The historical evolution of predictive modeling in healthcare has witnessed a transformative journey from basic statistical methods to the adoption of advanced machine learning algorithms, facilitating the in-depth analysis of complex electronic health records (EHRs) [1, 2]. The integration of EHRs has allowed researchers and policymakers to use more sophisticated statistical methods in predictive modeling and allowed for the potential to derive actionable insights [3, 4]. Such forecasting is important for healthcare systems to plan for enrollment, cost, and service utilization, particularly when introducing new services [5].

The Veterans Health Administration (VA) introduced chiropractic services in stages starting in Fiscal Year (FY) 2001, and since then has steadily expanded its chiropractic care delivery [1, 2]. This expansion has partly been driven by Congressional mandate [6], but largely reflects an organizational response to organic growth in the demand for non-pharmacological pain management strategies, especially given the high prevalence of musculoskeletal conditions among veterans [1]. This demand is paralleled by a significant body of evidence supporting key non-pharmacological treatments, including many delivered by chiropractors, as effective first-line treatments for highly prevalent musculoskeletal conditions such as low back pain and neck pain [7–9]. In VA in particular, there is preliminary evidence that opioid prescription use is lower after patients receive chiropractic visits than before [10].

As with all healthcare services, the VA provides chiropractic care via two primary mechanisms. One is “on-station” care, delivered in VA facilities by VA employees or contracted clinicians, and the other is via “Community Care,” which is care delivered in non-VA facilities, purchased from a Community Care Network (CCN) of providers [11]. CCN care is purchased for various reasons, including if services cannot be provided on-station within wait time and/or geographic distance standards.

Prior work has demonstrated significant increases in the use of VA chiropractic services through the on-station and CCN mechanisms [1, 2]. In Fiscal Year (FY) 2019, approximately 1.5% of VA patients received on-station chiropractic care and 1.7% received CCN chiropractic [1]. Yet this remains lower than key comparator populations. Between FY 2010–2015, the Department of Defense had a 13% utilization rate [12], while a 2023 National Health Interview Survey found that 11% of the general U.S. market used these services [13]. As the VA continues to expand its chiropractic care offerings, accurately estimating future trends in patient use and resource requirements is imperative.

Forecasting the growth of chiropractic services within the VA is essential for policy formulation, healthcare planning, and resource allocation. Accurate forecasts can help ensure that the VA continues to meet the evolving health needs of veterans, particularly those suffering from chronic pain and musculoskeletal conditions, which are prevalent in this population [14, 15]. Moreover, insights gained from these forecasts could potentially inform other healthcare systems that deliver chiropractic care.

This study aims to employ linear predictive modeling to forecast the utilization of chiropractic services and predict chiropractor clinical resource requirements within the VA over the next five years.

## Methods

### Study design and data sources

This was a serial cross-sectional analysis of national administrative data from the VA Corporate Data Warehouse, covering the fiscal years 2017–2022 (10/1/2016 to 9/30/2022). The study was designed and reported in accordance with the Strengthening the Reporting of Observational Studies in Epidemiology (STROBE) guidelines [16] for cross-sectional studies to ensure comprehensive and transparent reporting of observational research methods. The data included comprehensive records of healthcare service delivery within the VA system over this six-year observation period. We obtained data for VA chiropractic use through both the on-station and CCN mechanisms. The dataset was compiled between 15–23 August 2023, and the authors did not have access to information that could identify individual participants during or after data collection.

### Variables

The primary variables under investigation included the total number of healthcare users within the VA system and the subset of those utilizing chiropractic services. Three mutually exclusive cohorts were identified based on categories of chiropractic care received: on-station only; CCN only; and both, for patients having at least one visit in each of on-station and CCN.

Additionally, the analysis incorporated data on full-time equivalent (FTE) staffing numbers for chiropractors within the VA system and community care expenditure data.

### Statistical method

The five-year forecast period (FY2023–FY2027) was selected to provide a mid-range projection that allows for meaningful planning and resource allocation within the VA. The five-year horizon was chosen for its practical relevance, allowing stakeholders to anticipate short- to mid-term needs while considering the possibility of periodic model updates as more recent data becomes available.

Data analysis was performed using Microsoft Excel. Compound Annual Growth Rate (CAGR) calculations assessed the annual growth rates of unique outpatient users and chiropractic service utilizers.

A straight-line modeling approach was then applied to project utilization for fiscal years 2023 to 2027. This method, commonly used in healthcare forecasting when projecting service utilization trends, has been effectively employed in studies forecasting patient visits in hospital settings [17]. This analysis included disaggregation by care modality (on-station, CCN, and Both).

For staffing projections, the average number of unique patients per chiropractor FTE observed during the study period was calculated. This average was used to forecast the number of FTEs required to meet future service demands. Similarly, the CAGR for the average cost per unique patient receiving CCN chiropractic services was calculated and used to forecast future expenditures based on identified growth trends.

### Description of Compound Annual Growth Rate (CAGR)

CAGR is calculated using the formula:

$$CAGR={\left(\frac{End\;Value}{Start\;Value}\right)}^{\frac{1}{Number\;of\;Years}}-1$$

Where End Value is the final number of users at the end of the period, Start Value is the initial number of users at the beginning of the period, and Number of Years is the total number of years in the period.

### Ethical considerations

This study was approved by the institutional review board (IRB) of the Veterans Affairs Connecticut Healthcare System (IRB# 1713505–1). It has been granted a waiver of informed consent and is compliant with the Health Insurance Portability and Accountability Act (HIPAA).

## Results

### Historical growth and utilization of chiropractic services in the VA

Over the six-year observation period, the overall utilization rate of VA chiropractic services increased from 1.4% in FY 2017 to 3.5% in FY 2022, representing a CAGR of 20.2%. Chiropractic services exclusively through the CCN grew at a CAGR of 25.1%, while on-station grew at a CAGR of 13.5%. Veterans who received at least 1 visit through both CNN and on-station care mechanisms (categorized as “both”) saw the most growth with a CAGR of 30.0%.

### Projected growth and utilization of chiropractic services in the VA

**Fig 1:**
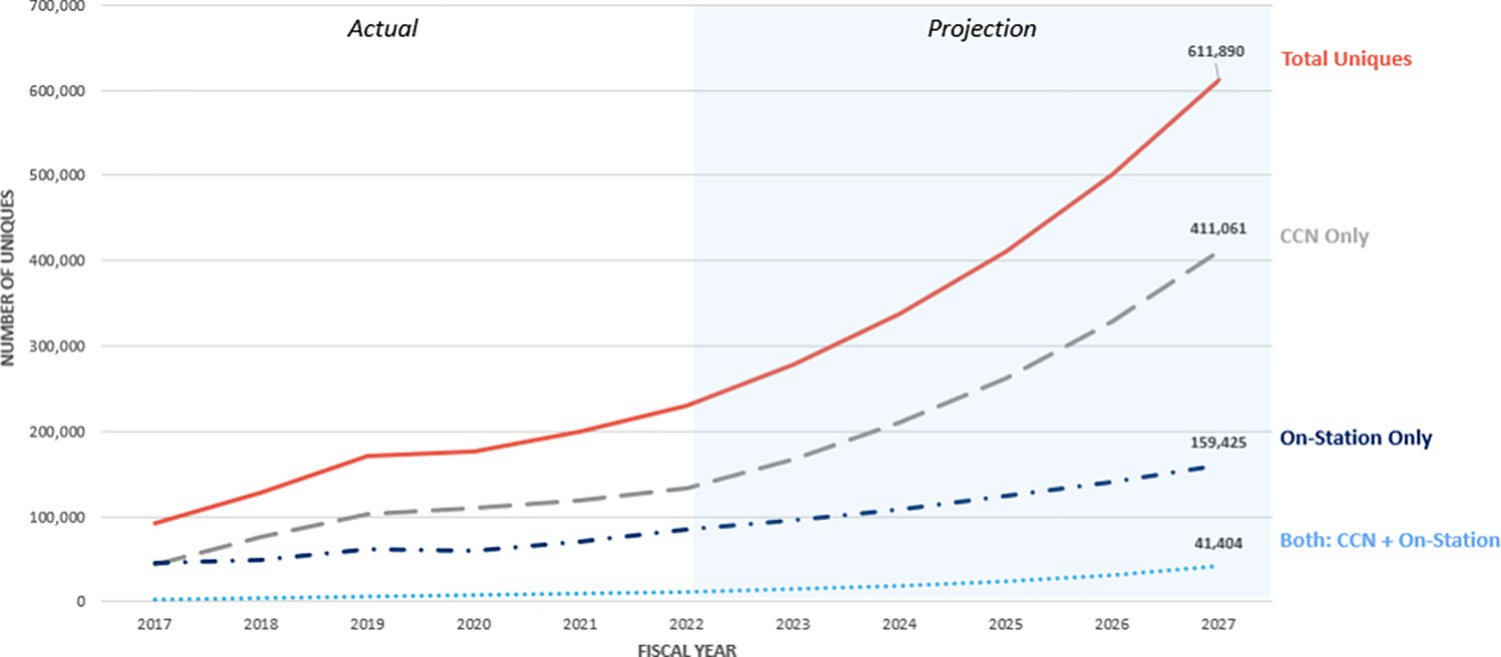
Projected growth of chiropractic service utilization by mode (FY 2022–2027).

The forecast for chiropractic service utilization within the VA for FY 2023 through FY 2027 predicts that by FY 2027, the total number of veterans utilizing any mode of chiropractic service will reach 611,890 (Fig 1). The projections for each delivery mechanism—CCN, on-station, and both—were modeled using the historical CAGRs of 25.1%, 13.5%, and 30.0%, respectively. This reflects the potential of a 167% increase across all modes of utilization from FY 2022 to FY2027. Veterans exclusively using CCN services are projected to reach 411,061 by FY 2027, with on-station care at 159,425 and both at 41,404.

**Fig 2:**
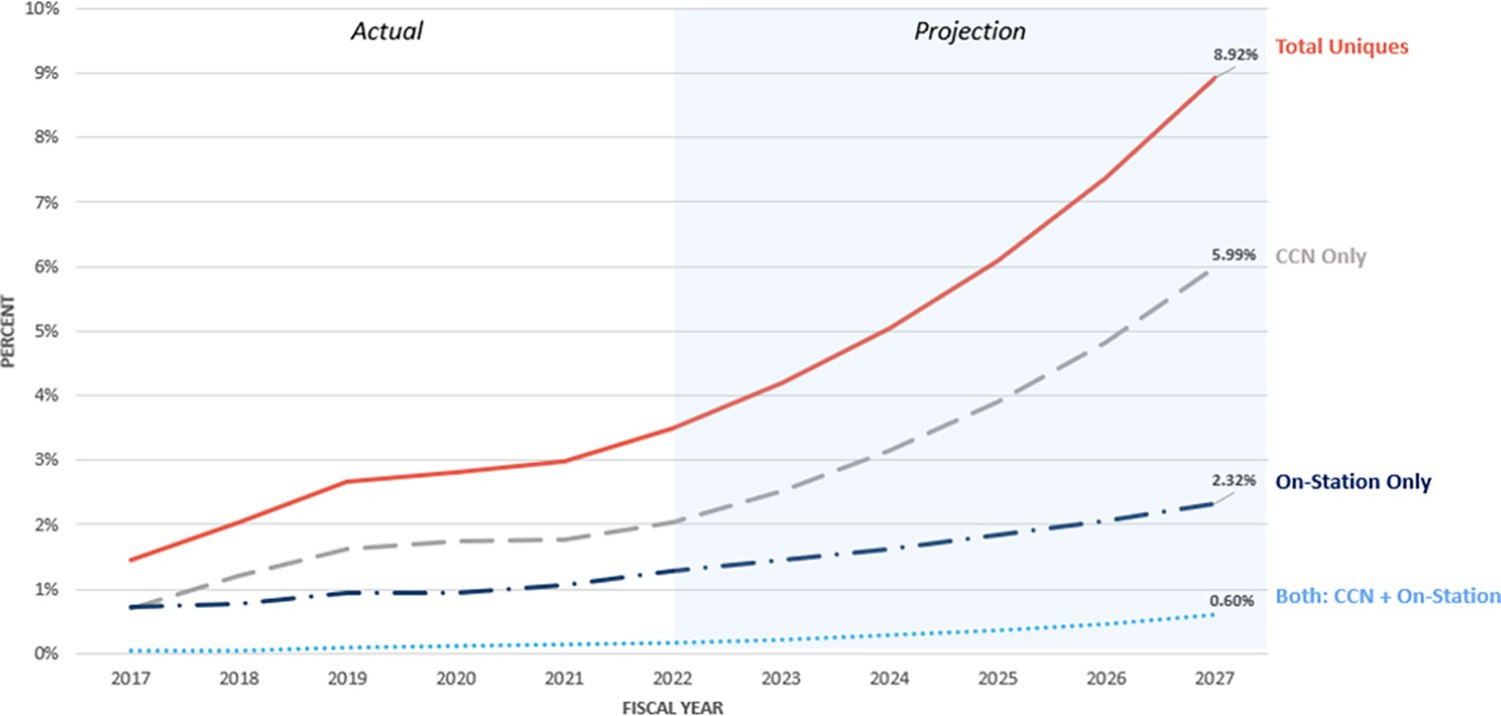
The proportion of VA users utilizing chiropractic services (FY 2022–2027).

In FY 2022, 3.5% of VA patients utilized chiropractic care services. By FY 2027, this proportion is projected to reach 8.9% (Fig 2). The proportion of VA patients exclusively using CCN services is expected to increase from 2.0% in FY 2022 to 5.9% in FY 2027. Similarly, on-station care is projected to rise from 1.3% to 2.3% and the cohort of “both” is projected to rise from 0.2% to 0.6%.

### Chiropractic workforce

**Fig 3:**
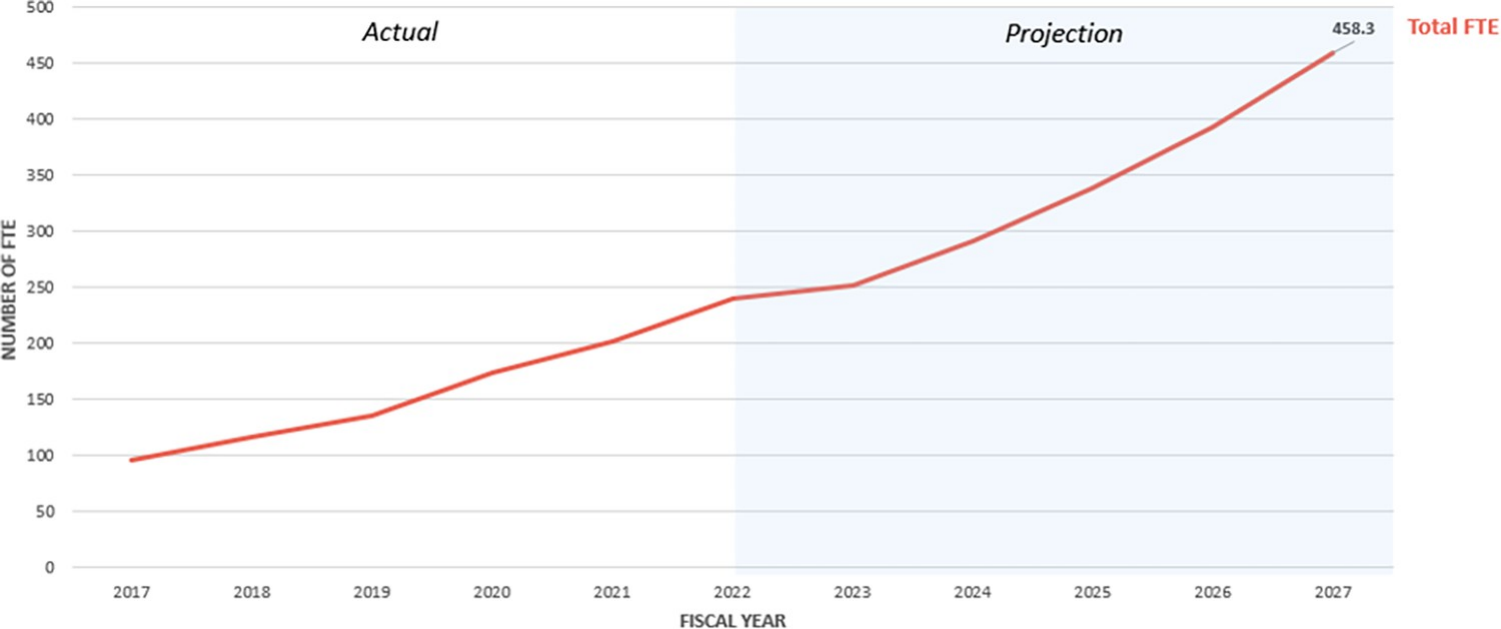
Projected staffing needs for on-station chiropractors.

For each full-time equivalent (FTE) of chiropractic clinical effort, VA provided on-station care to an average of 438 unique veterans per year between FY 2017 and FY 2022. To meet the projected demand of delivering on-station care to 200,829 unique veterans by FY 2027, at least 458 chiropractic clinical FTE would be required, assuming current productivity and service delivery parameters remain constant. This projection is does not account for addressing any potential unmet demand, excessive wait times, or innovations in access to care. As such, this estimate reflects the minimum staffing level needed to achieve the projected service delivery (Fig 3).

### Projected community care expenditure

The average cost per veteran receiving CCN chiropractic services grew at a CAGR of 3.8% annually from FY 2017 to FY 2022, with a forecasted cost per veteran of $1,223 in FY 2027. With the projected growth in CCN utilization, 411,061 veterans are forecasted to use CCN chiropractic care by FY 2027 at an estimated cost of $553.4 million (Table 1).

**Table 1:** Historical community care expenditures (FY2017-2022).

Fiscal Year	2017	2018	2019	2020	2021	2022
**Total CCN Expenditures**	$38,910,495	$77,655,778	$95,975,404	$106,687,899	$124,266,909	$146,578,776
**Average Patient Cost**	$836	$975	$877	$907	$968	$1,012

## Discussion

To our knowledge, this study presents the first effort to forecast future chiropractic service use in the largest US healthcare system. Our projections depict continued growth in VA chiropractic use, by both the on-station and CCN mechanisms, up to a total of 8.9% of VA healthcare users receiving VA chiropractic care by 2027. This trend aligns with the broader healthcare trends towards evidence-based non-pharmacological pain management strategies [7] and highlights the VA’s role in addressing veterans’ musculoskeletal healthcare needs [18].

Musculoskeletal conditions remain a leading cause of disability among veterans [19], with chronic pain significantly impacting their quality of life and healthcare utilization [20]. The VA provides care for a high prevalence of musculoskeletal disorders (MSDs), including low back and neck pain [21]. Outside of the VA system, chiropractic care is commonly used for these conditions. In studies of US commercial insurance and Medicare Advantage populations, chiropractors were the entry point providers for 23.1% of new treatment episodes for low back pain, second to primary care physicians at 53.0% [22]. For new treatment episodes for neck pain, chiropractors were the most common initial provider, accounting for 45.2% of cases, with primary care physicians second at 33.4% [23].

Furthermore evidence suggests that chiropractic care can lead to meaningful reductions in opioid use [24–26], a critical outcome given the risks of long-term opioid therapy. For instance, a recent study utilizing data from the Arkansas All Payer’s Claims Database demonstrated that individuals receiving chiropractic care for non-cancer spinal pain had a 12% lower likelihood of initiating opioid therapy and a 44% reduced likelihood of long-term opioid use [26]. Lastly in 2024 a systematic review and metanalysis also demonstrated that the rate of opioid prescription fills decreased by 34%, and refills were 73% lower among chiropractic care recipients [27]. These findings reinforce the importance of expanding access to non-pharmacological treatments like chiropractic care for effective musculoskeletal pain management within the veteran population.

The increase in VA chiropractic service utilization predicted by our results would put VA closer to the range of 11–14% population use reported in the Department of Defense and private US healthcare systems [12, 13, 15]. However, our results show that through 2022 VA has been providing lower access to chiropractic care than these systems, and by 2027 it will likely still be lagging. Due to competing priorities, budgetary considerations, and the ongoing evolution of chiropractic care’s acceptance in mainstream healthcare systems [28–30], the actual use of VA chiropractic care in the future may vary substantially.

## Limitations

Our projections assume stability in VA budget, healthcare priorities, and the prevailing acceptance of chiropractic care; however, changes in these could affect model accuracy. We did not account for the possibility that as on-station access increases, some patients might switch from CCN to on-station, which would increase the needed on-station resources. We did not attempt to assess the additional administration burden, space, or other logistic resources that would be needed to support the expanded use of chiropractic care. Our model did not consider potential changes in the degree to which veterans use VA healthcare as opposed to other options (eg private insurance, self-pay) available to them. Significant changes in the size of the VA healthcare user population would alter our projections, as would change to the typical rate of chiropractic of visits per patient per year. Future work should employ more sophisticated actuarial approaches to include these factors.

## Conclusion

Use of VA chiropractic care has grown to 3.5% of the VA healthcare user population by the end of FY 2022 and is projected to reach 8.9% of that population by the end of FY 2027. This work provides preliminary results that can inform strategic resource planning supporting the anticipated increased use of both on-station and CCN chiropractic services.
